# Modeling Urban Hydrology and Green Infrastructure Using the AGWA Urban Tool and the KINEROS2 Model

**DOI:** 10.3389/fbuil.2018.00058

**Published:** 2018

**Authors:** Yoganand Korgaonkar, D. Phillip Guertin, David C. Goodrich, Carl Unkrich, William G. Kepner, I. Shea Burns

**Affiliations:** 1 School of Natural Resources and the Environment, University of Arizona, Tucson, AZ, United States; 2 Agricultural Research Service, Southwest Watershed Research Center, USDA, Tucson, AZ, United States; 3 Office of Research and Development, US Environmental Protection Agency, Las Vegas, NV, United States

**Keywords:** urban hydrology, green infrastructure, semi-arid, hydrologic model, stormwater, AGWA, KINEROS2, GIS

## Abstract

Urban hydrology and green infrastructure (GI) can be modeled using the Automated Geospatial Watershed Assessment (AGWA) Urban tool and the Kinematic Runoff and Erosion (KINEROS2) model. The KINEROS2 model provides an urban modeling element with nine overland flow components that can be used to represent various land cover types commonly found in the built environment while treating runoff-runon and infiltration processes in a physically based manner. The AGWA Urban tool utilizes a Geographic Information System (GIS) framework to prepare parameters required for KINEROS2, executes the model, and imports results for visualization in the GIS. The AGWA Urban tool was validated on a residential subdivision in Arizona, USA, using 47 rainfall events (June 2005 to September 2006) to compare observed runoff volumes and peak flow rates with simulated results. Comparison of simulated and observed runoff volumes resulted in a slope of 1.00 for the regression equation with an R^2^ value of 0.80. Comparison of observed and simulated peak flows had a slope of 1.12 with an *R*^2^ value of 0.83. A roof runoff analysis was simulated for 787 events, from January 2006 through December 2015, to analyze the water availability from roof runoff capture. Simulation results indicated a 15% capture of the average monthly rainfall volume on the watershed. Additionally, rainwater captured from roofs has the potential to provide for up to 70% of the domestic annual per capita water use in this region. Five different scenarios (S1 - base, S2 - with retention basins, S3 - with permeable driveways, S4 - with rainwater harvesting cisterns, and S5 - all GI practices from S2, S3, and S4) were simulated over the same period to compare the effectiveness of GI implementation at the parcel level on runoff and peak flows at the watershed outlet. Simulation results indicate a higher runoff volume reduction for S2 (53.41 m^3^ average capacity, average 30% reduction) as compared to S3 (average 14% reduction), or S4 (3.78 m^3^ capacity, average 6% reduction). Analysis of peak flows reveal larger peak flow reduction for S2. S3 showed more reduction of smaller peak flows as compared to S4.

## INTRODUCTION

Urbanization causes an increase in impervious surfaces (e.g., roofs, driveways, parking lots, and roads) by replacing vegetation and pervious natural areas. As a result, the area available for the infiltration of rainwater is substantially reduced. Additionally, soil compaction due to subdivision construction can result in reduced pore-water storage capacity thereby reducing infiltration rates (Gregory et al., 2006; Pitt et al., 2008; Woltemade, 2010; Yang and Zhang, 2011; Kennedy et al., 2013). Surface runoff volumes and peak discharges can increase significantly because of reduced infiltration and increases in connected impervious areas (Shuster et al., 2005). Increased surface runoff can also result in higher downstream loads of urban pollutants, such as lead, zinc, iron, suspended solids, fecal coliform bacteria, nitrogen, phosphorus, and hydrocarbons (Duda et al., 1982; Characklis and Wiesner, 1997; Norman et al., 2008; Bedan and Clausen, 2009).

Green Infrastructure (GI, also referred to as Low Impact Development, or LID) has gained prominence in the management of stormwater to mitigate the effects of urbanization on watershed hydrology. There has been a shift in stormwater management techniques from traditional practices, such as curb-and-gutter systems, large detention basins, and hardened channels, to source control measures that use a variety of cost effective on-site design techniques to store, infiltrate, evaporate, and detain runoff. These practices include rain gardens, bioretention cells or basins, permeable pavements, green roofs, swales, infiltration trenches, roof runoff harvesting, and impervious disconnection (Dietz, 2007) that have varying degree of performance and effectiveness (Hopton et al., 2015).

Analysis and mitigation of urban water quantity and quality issues, as well as the role of GI, requires a better understanding of the physical hydrological processes within urban areas. Various urban hydrological models have been successful in representing and simulating urban hydrological processes as well as GI practices (Zoppou, 2001; Elliott and Trowsdale, 2007; Jayasooriya and Ng, 2014). All of these models have inherent assumptions, with a goal to represent hydrological processes accurately, while keeping the model simple and easy-to-use. Effective urban hydrological models are typically spatially distributed as they attempt to represent the heterogeneous nature of urban landscapes. By their nature, spatially distributed hydrologic models require copious spatial data on urban landscapes and hydrologically important watershed characteristics (e.g., land cover, land use, GI, soils, and topography). The process of acquiring this data and extracting model parameters can be tedious and lengthy. Geographic Information Systems (GIS) provide capabilities and techniques to efficiently address these tasks, and visualize, analyze, and interpret spatial patterns (DeVantier and Feldman, 1993; Stuart and Stocks, 1993; Sui and Maggio, 1999; Vieux, 2001; Goodchild et al., 2005). The increasing availability of spatial data coupled with GIS capabilities, affords greater opportunity to simplify the process of using this data in hydrological models. Numerous hydrological models have been coupled with GIS to facilitate parameter extraction from spatial data and visualization of modeling results (Srinivasan and Arnold, 1994; Bhaduri et al., 2000; Miller et al., 2007; Chen et al., 2009; Lee et al., 2012).

The US Department of Agriculture’s (USDA) Agricultural Research Service (ARS) developed the Kinematic Runoff and Erosion (KINEROS2) model. KINEROS2 is a spatially distributed, physically based, event driven model that simulates runoff and erosion for small watersheds (Smith et al., 1995; Goodrich et al., 2012). Overland flow is simulated using kinematic wave equations over rectangular elements with linear or curvilinear hillslopes, and concentrated flow in trapezoidal channels. Infiltration is simulated using a modified Smith-Parlange infiltration model (Parlange et al., 1982). KINEROS2 contains a rectangular urban element that consists of up to nine overland flow areas that contribute to one-half of a paved, crowned street. These nine overland flow areas include: (1) directly connected pervious (DCP) area, (2) directly connected impervious (DCI) area, (3) indirectly connected impervious (ICI) area, (4) indirectly connected pervious (ICP) area, (5) connecting pervious (CP) area, (6) connecting impervious (CI) area, (7) non-contributing (NC) area, (8) an infiltrating retention basin (RB) area, and (9) street half on to which the aforementioned overland flow areas contribute runoff. All nine flow areas are not required for each urban element for simulation.

A single housing parcel in the urban area can have various surfaces that can affect hydrology differently. These surfaces include impervious roofs and driveways, pervious yards, and water sinks that can pond during rainfall events. With the help of the nine flow areas, the urban element can represent various flow-on/flow-off processes from these surfaces. These flow areas allow for model representation at a finer scale allowing better understanding of hydrological interactions and effects at a housing parcel level. The urban element in KINEROS2 can be used to represent a single housing parcel or a number of parcels in an urban development (Kennedy et al., 2013). The ICI can be used to represent roofs, DCI for driveways, CP for front yards and DCP for all other yards, NC for swimming pools or walled areas, and RB for retention basins or rain gardens on the parcels. A typical urban watershed can be represented as a series of urban elements with the assumption that runoff flows from each element into a street or alley half and follows the path along the street or alley to the watershed outlet.

KINEROS2 requires parcel parameters in the form of dimensions, slope and fractional area of the urban overland flow areas; and street parameters in the form of width, grade, and cross slope. KINEROS2 also requires land cover parameters, which include values for hydraulic roughness for streets, impervious and pervious surfaces, interception depths for impervious and pervious surfaces, and canopy cover fractions. Additionally, KINEROS2 requires soils parameters in the form of saturated hydraulic conductivity (Ks), coefficient of variance of Ks to represent small-scale infiltration variability (Smith and Goodrich, 2000), mean capillary drive (potential of capillary suction in unsaturated soils), porosity, pore size distribution index, and volumetric rock fraction. Precipitation data in the form of time-intensity or time-accumulated depth pairs drives the KINEROS2 model. An urban simulation using KINEROS2 yields output in the form of runoff, infiltration, and storage for each individual element. Additionally, KINEROS2 can also output peak flow hydrographs at each element and at the outlet. The KINEROS2 urban element assumes negligible sediment production and therefore does not output sediment yield in this version.

KINEROS2 was used to model runoff from a small residential development in Sierra Vista, Arizona in studies conducted by Kennedy (2007) and Kennedy et al. (2013). Kennedy et al. (2013) looked at three different discretization levels by lumping the 66 residential lots into 1, 5, and 23 KINEROS2 urban elements, and concluded that the accuracy of the model increased with the level of detailed representation of the development. Kennedy (2007) also performed a sensitivity analysis that indicated saturated hydraulic conductivity to be the primary estimation parameter to simulate runoff behavior. The study also highlighted the minimal uncertainty of parameter estimates in the urban watersheds, concluding that model uncertainty arose from model structural errors and input data error. Yatheendradas et al. (2008) performed a comprehensive sensitivity analysis on the KINEROS2 model for flash flood forecasting, and concluded that the predominant source of uncertainty in modeled runoff response is due to depth/volume bias in rainfall estimates, followed by the saturated hydraulic conductivity, soil volumetric rock fraction and soil hydraulic roughness. The capabilities of KINEROS2 to model urban hydrology in detail forms the basis of the present study by representing each parcel in the watershed using the urban element.

The Automated Geospatial Watershed Assessment (AGWA) tool was jointly developed by the USDA-ARS, the University of Arizona, and the EPA/ORD (Miller et al., 2007). AGWA is a GIS-based tool that uses existing spatial datasets in the form of digital elevation models (DEM), land cover maps, soil maps and weather data to prepare parameters for hydrological models. Currently, AGWA supports the Soil and Water Assessment Tool (SWAT; Arnold et al., 1998), the KINEROS2 model, and the Rangeland and Hillslope Erosion model (RHEM; Nearing et al., 2011). AGWA computes and supplies the parameters to these models, runs the models, and imports the results back in the GIS for visualization and analysis. AGWA is designed to provide qualitative estimates of runoff and erosion relative to landscape change. It, like virtually all watershed models, cannot provide quantitative estimates without careful calibration using high quality rainfall-runoff observations.

AGWA has been used to assess land use impacts on water resources in a number of studies (Hernandez et al., 2000; Miller et al., 2002; Baldyga et al., 2004; Kepner et al., 2004, 2009, 2012; Hamad et al., 2012; Nazarnejad et al., 2012; Baker and Miller, 2013; Burns et al., 2013; Barlow et al., 2014). Several studies to assess post-fire hydrological responses in watersheds have been conducted using AGWA (Canfield et al., 2005; Goodrich et al., 2005; Sidman et al., 2016). Other applications of AGWA include rangeland management (Goodrich et al., 2011; Weltzet al., 2011), flood hazard studies (Norman et al., 2010; Nedkov and Burkhard, 2012) and watershed assessment (Abdulla and Eshtawi, 2007; Yang and Li, 2011).

This study presents a GIS approach to simulating urban hydrology and GI using the KINEROS2 model, and its integration with the AGWA tool. This study has three objectives: (1) Validate the capabilities of the AGWA Urban tool and KINEROS2 to model urban hydrology and green infrastructure; (2) Analyze the roof runoff availability for water use in a semiarid climate; and, (3) Analyze the effectiveness of GI practices on flood mitigation by reducing runoff volumes and peak flows at the watershed outlet.

## METHODS

### Study Area

**FIGURE 1 |:**
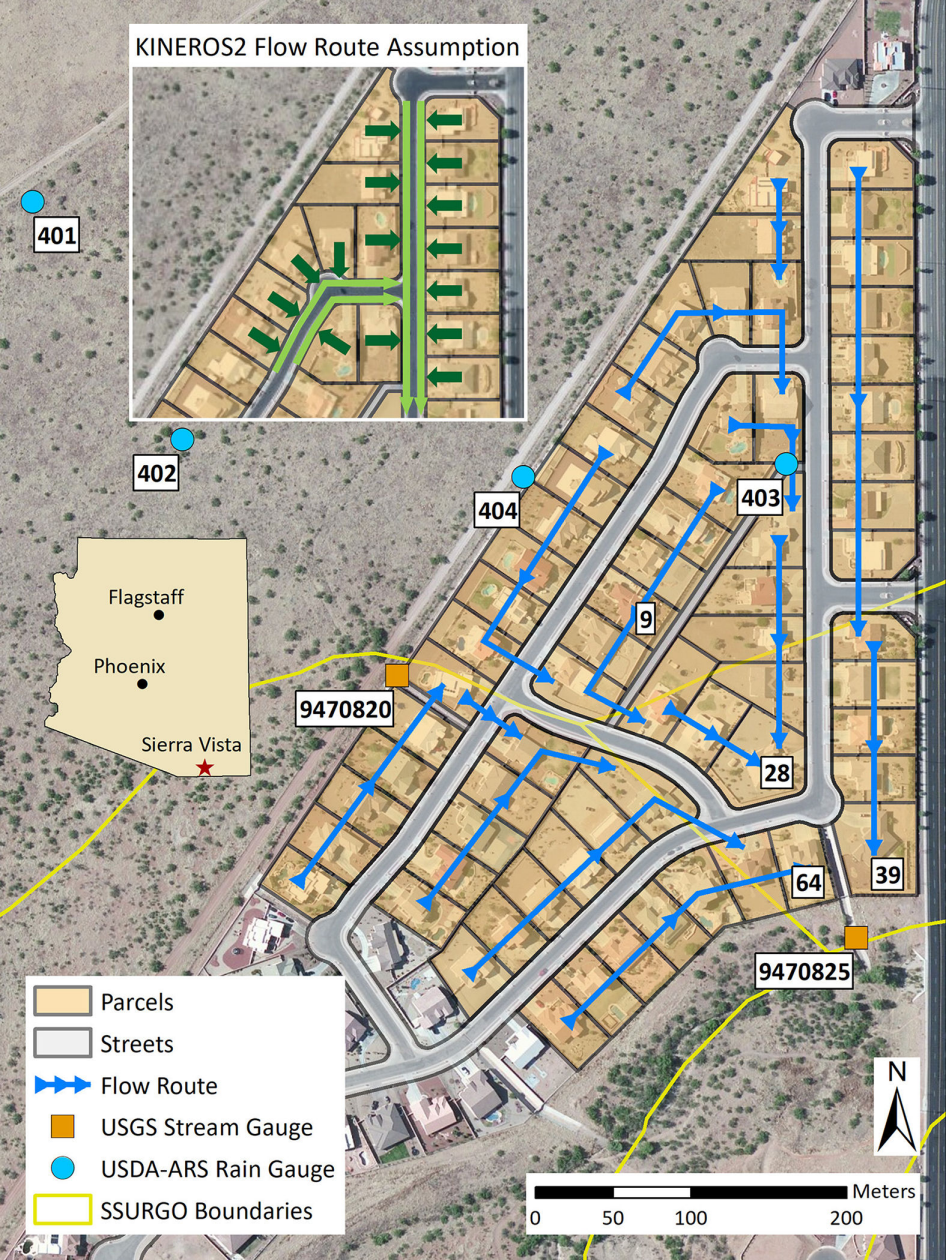
The La Terraza urban watershed in Sierra Vista, Arizona. Inset: KINEROS2 assumption of the user-defined flow route. KINEROS2 assumes that overland flow is off the parcel into the street (dark green arrows), and accumulates along the street toward the watershed outlet (light green arrows).

The La Terraza subdivision in Sierra Vista, Arizona (Figure 1) was selected as the study area based on Kennedy et al. (2013) study, and the availability of input datasets and high quality observations of rainfall and runoff. Sierra Vista is located in Cochise County in southeastern Arizona, at an elevation of approximately 1,300 m, with an annual average precipitation of 360 mm and annual mean temperature of 17.4°C based on records over the period 1981–2000. Sierra Vista is located west of the San Pedro River and is contained in the Upper San Pedro HUC 15050202 watershed. The La Terraza subdivision is a residential development spanning 14 hectares located in the western part of the city. This study focused on an urban watershed consisting of 66 housing lots with an average parcel size of 1,780 m^2^, average house area of 380 m^2^, average driveway area of 108 m^2^, and 7.3m wide asphalt streets within the La Terraza subdivision. An undeveloped upland grassland watershed to the west contributes runoff to the urban watershed via a concrete channel. Runoff within the urban watershed flows over the streets, with the exception of a corrugated pipe that is located underneath La Terraza Drive that drains a 1.3 ha area in the northern part of the urban watershed to the outlet. Runoff exits at the outlet via another concrete channel to the southeast of the urban watershed.

### AGWA Urban Tool

The AGWA Urban tool was designed and developed to harness the capabilities of the KINEROS2 model to represent urban areas in detail, and to create tools to use spatial datasets in parameter preparation for the model. The AGWA Urban tool was developed using the .NET Framework using the C# and VB.NET programming languages in the Microsoft Visual Studio Integrated Development Environment. ESRI provides an ArcObjects software development kit for the .NET Framework to build Windows applications with GIS functionalities. Using .NET and ArcObjects, Windows-based forms were developed that interface with existing GIS functionalities in ESRI ArcMap. The AGWA Urban tool is an ArcMap add-in that provides tools to prepare parameter files for the KINEROS2 model, runs the model, and imports the results into ArcMap for spatial visualization and analysis. Input parameters are generated from parcel, street, land cover, soils, and precipitation datasets. Additionally, inputs in the form of overland flow paths, and GI designs and locations can be manually provided. Each parcel is represented as a KINEROS2 urban element. The AGWA Urban tool executes the KINEROS2 model based on these input parameters. Runoff and infiltration results are visualized as maps and peak flow results are displayed as hydrographs.

### Steps Involved in an AGWA Urban Simulation

#### Setup urban geodatabase

The AGWA Urban tool prepares the simulation workspace with a geodatabase to store spatial and tabular data prepared and used by an urban simulation. This step requires spatial data for the parcels and the streets. Required inputs are parcel and street maps that contain location data and dimensions (length, width, house size, driveway size, and slope) for each of the parcels and streets. The subsequent steps create files and relational tables that are stored in the simulation workspace and geodatabase, respectively.

#### Flow routing

Overland stormwater flow paths are required as inputs in the flow routing step. These flow paths can be input as map layers, or can be created using the drawing tools provided by ArcMap. The AGWA urban tool links the flow paths to the parcels, performs checks to ensure continuity in the paths, and creates a table representing the flow route for the urban watershed.

#### Parameterization

Parcel, street, land cover, and soils parameters are defined in the parameterization step. Parcels and street parameters can be defined based on existing data for each parcel (provided via the parcel map), or created homogeneously for all parcels. Similarly, land cover parameters can be created for all parcels. The AGWA Urban tool requires the nationally available Soil Survey Geographic Database (SSURGO) to extract soil parameters based on the input soils map. All parameters created in this step are stored in a parameterization table that maybe modified to reflect variations in parameters for each parcel. Note that SSURGO soils data are typically derived for a non-developed environment. Soil importation and compaction for subdivision and foundation pad construction may result in soil parameters substantially different from those represented in the SSURGO soils database (Kennedy et al., 2013). In this case, local, post-construction soil hydraulic property measurements are recommended.

#### GI design and placement

Currently the AGWA urban tool provides options to design three different kinds of GI practices: (1) retention basins, (2) permeable driveways, and (3) rainwater harvesting cisterns. For retention basins, the size and depth of the basin are required, along with the hydraulic conductivity of the basin material. Hydraulic conductivity values for the driveways are required in order to model the driveways as permeable. To use the rainwater harvesting functionality, the volume of the capturing cistern is required. After these designs are created, they can then be applied to the parcels individually or collectively.

#### Precipitation

The AGWA Urban tool accepts design storm precipitation data through the National Oceanic Atmospheric Administration (NOAA) Atlas 14-point precipitation frequency estimates databases, or user-defined depths and hyetographs, and converts them into a time-depth format required by KINEROS2. KINEROS2 also requires an initial soil saturation value that can be by rain gage, by element, or for the entire watershed. The AGWA Urban tool creates a precipitation text file based on the user inputs in the simulation workspace.

#### KINEROS2 input files

KINEROS2 requires ASCII text files as inputs for model execution. The AGWA Urban tool converts the spatial and tabular data from the preceding steps into text files and saves them in the simulation workspace. A parameter file stores the input parameter data for each parcel and the sequence of execution of the parcels. A KINEROS2 control file directs the model with the parameter filename, precipitation filename, output filename, duration, and time steps for the simulation. A GI volumes file is created with initial volumes of the cisterns, and retention basins, for each parcel. The volumes in this file are updated after the simulation. A batch file is created that can execute the KINEROS2 model on the command line. Multiple simulations based on different combinations of flow routing, parameterization, and precipitation data can be created in this step. The AGWA Urban tool also provides an optional batch mode option to prepare and run KINEROS2 for multiple precipitation events.

#### Model execution

KINEROS2 is a FORTRAN based model that runs on the Microsoft command line. The AGWA Urban tool executes the KINEROS2 model using the created input files. A command prompt displays the progress of the simulation and its status as success or if it encountered any errors. KINEROS2 creates an output text file, which summarizes water balances for each urban element and the entire watershed. Hydrograph files are also created for each parcel and the entire urban watershed.

#### Importing and visualizing results

The AGWA Urban tool imports text file results from KINEROS2 simulations into ArcMap and links them to the spatial data, thereby allowing the user to visualize the data using the GIS interface. The user can visualize runoff and infiltration volumes for each individual parcel, as well as accumulated runoff along the streets as stormwater flows toward the outlet. Additionally, the user can also compare runoff, infiltration, and accumulated runoff between simulations using absolute and percent change options. Hydrographs can be viewed at the outlet of the watershed as well as for each individual parcel.

### AGWA Urban Tool Validation

The AGWA Urban tool was validated using forty-seven observed rainfall and runoff events from July 2005 through September 2006 for the La Terraza urban watershed. During this analysis, there were no GI practices installed on any of the parcels. Hence, GI was not included in the validation analysis. Rainfall data was extracted from four recording rain gauges (USDA Southwest Watershed Research Center, SWRC, gauges 401, 402, 403, and 404 - data available at https://www.tucson.ars.ag.gov/dap/; Figure 1) with areal average rainfall event totals ranging from 2 to 35 mm (events less than 2 mm were not used). Runoff, both in and out of the La Terraza urban watershed, was measured by v-notch weirs (U.S. Geological Survey, USGS, weirs 9470820 and 9470825, respectively; Figure 1). Because peak and runoff values are unreliable when the outlet “v” notch section of the weir is overtopped, events that met this criterion were excluded to provide a high-quality data set of 47 events.

The AGWA Urban tool was used to setup the input parameters for the model. Parcel and street files were obtained from the Cochise County’s Information Technology department. Parcel dimensions and street widths were extracted from these files. Base map imagery, available in ArcMap, was used to determine the house and driveway areas on each parcel manually. Every parcel was assumed to have a front yard area and a noncontributing area equal to 10% of the total lot area. Values for slope, and land cover parameters were obtained from Kennedy et al. (2013). Soil parameters were obtained using the SSURGO database. The SSURGO dataset identified three different soil map units within the La Terraza urban watershed. Each parcel was assigned a specific set of soils parameters based on the soil map unit it intersected. Flow paths were drawn to represent the actual overland stormwater route converging toward three parcels (IDs 28, 39, 64) toward the southern part of the urban watershed (Figure 1). The adder element in KINEROS2 was utilized to combine these three parcels to represent the outlet. Stormwater was assumed to flow off the lots into the streets and along the streets to the outlet. This setup of modeling elements with associated parameters is hereafter referred to as the validation configuration. Runoff from the upland grassland watershed was provided to the model using the injection functionality available in KINEROS2. The parameter text file created by the AGWA Urban tool was modified to incorporate an injection element. The AGWA urban tool created input files for each of the events, and executed the model 47 times. Every event had a measured initial soil saturation observation associated with each rain gage. Each simulation was run for a duration of 720 min with a time step of 30 s. Output runoff volumes and peak flows were then extracted for analysis.

### Roof Runoff Analysis

Each parcel in the above validation configuration was fitted with a virtual 37.85 m^3^ (10,000 gallon) cistern to estimate potential harvesting capacity, with the assumption that the cistern was emptied before every event. The cistern capacity was chosen in order to ensure that all roof-generated runoff was captured by the cistern, thereby simulating maximum roof runoff capture. Rainfall data for this analysis was extracted from SWRC Gauge 403 for the period ranging from January 2006 to December 2015, comprising 787 rainfall events. The analysis was run with a 1-min time step for each of the rainfall events in the 10-year period. The AGWA urban tool was used to design and place the cisterns on each of the parcels. The batch mode functionality was utilized to write the input files and execute the model for each of the events. Post-simulation cistern volumes for each parcel were extracted and compiled for roof runoff analysis.

### Case Study Scenarios

Five scenarios (Table 1) were designed to assess the impacts of various GI practices in the urban watershed. Scenario 1 (S1) is considered as the base scenario, where the validated configuration, described in section AGWA Urban Tool Validation, was simulated over the analysis period of 10 years without any GI practices. In scenario 2 (S2), a retention basin (RB) was installed in each parcel in the validation configuration. The retention basin was designed with a surface area equal to 10% of the total parcel area, a basin depth of 0.3 m, and a hydraulic conductivity of 8.3 mm/h. The retention basin was assumed empty at the start of each rainfall event. Note that runoff from driveways is captured by the retention basins to simulate retention of all available runoff from the parcel. Scenario 3 (S3) converted all driveways in the validation configuration to permeable driveways (PD) with a hydraulic conductivity of 8.3 mm/h. In scenario 4 (S4), each of the parcels in the validation configuration was installed with a 3.78 m^3^ (1,000 gallon) cistern to simulate rainwater harvesting (RH) off the roofs. The cistern was assumed empty at the start of each rainfall event. For scenario 5 (S5), GI designs from S2, S3, and S4 were all installed on each parcel in the validation configuration. The same 787 events from the roof runoff analysis were used to simulate these scenarios. Initial soil saturation was assumed as 0.2 for all events. The AGWA Urban tool was used to design and place the different GI practices on each lot, to create the input files, and execute the KINEROS2 model using the batch mode functionality. Runoff and peak flow results at the outlet of the urban watershed were extracted and compiled for analysis.

## RESULTS

### The AGWA Urban Tool

**FIGURE 2 |:**
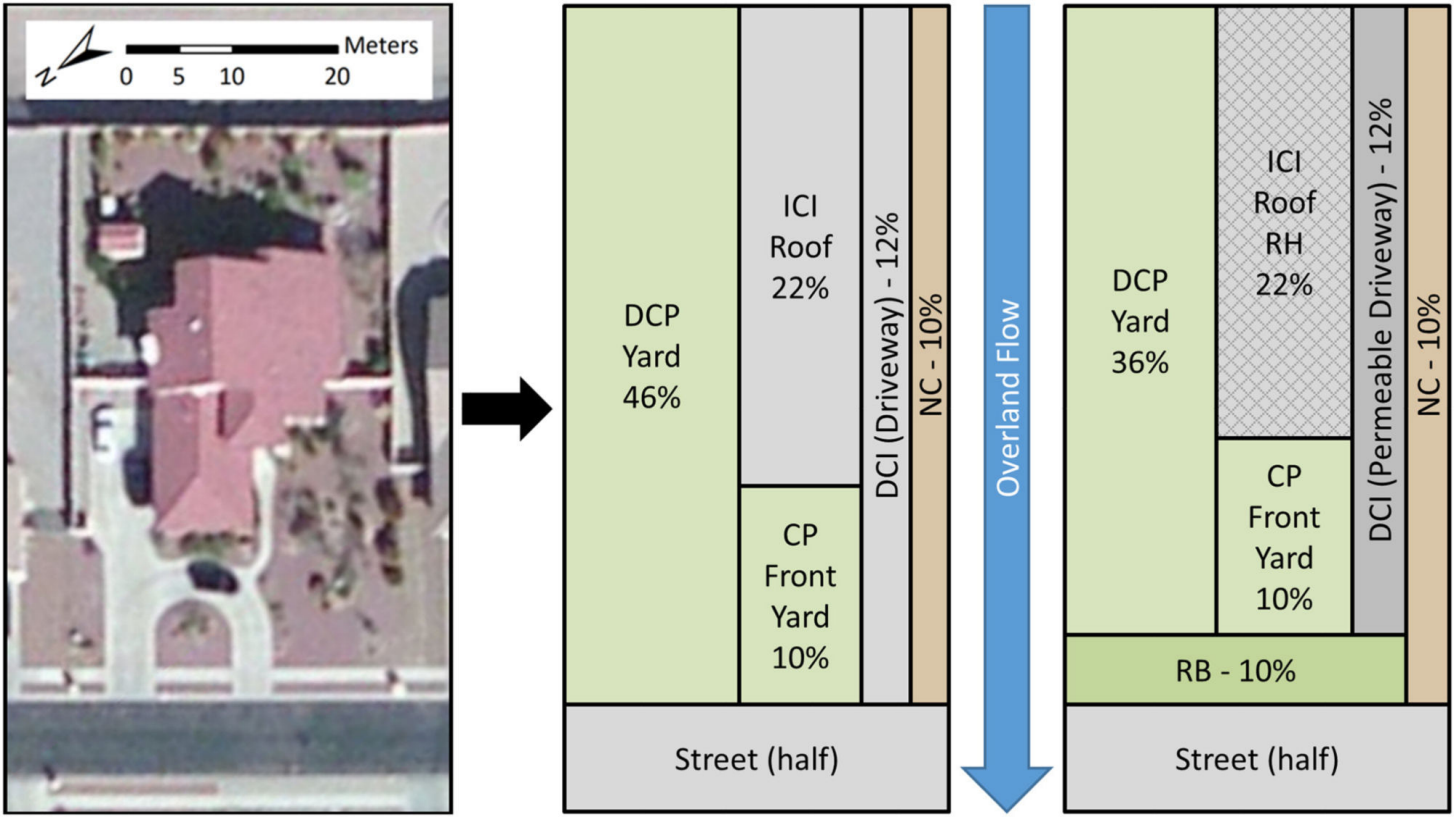
Parcel ID 9 in the La Terraza urban watershed **(Left)**, KINEROS2 representation without GI **(Center)**, and with retention basin (RB), permeable driveway (PD), and rainwater harvesting (RH) GI practices **(Right)**. Percent values of each of the overland flow areas are indicative of the percent of the total parcel area. DCP, directly connecting pervious; ICI, indirectly connected impervious; CP, connected pervious; DCI, directly connected impervious; NC, noncontributing area.

The AGWA Urban tool provides an easy-to-use framework to setup and execute the KINEROS2 model. Parcel ID 9 (Figure 1) and its representation using the urban element with and without GI practices, based on fractional areas of roofs, driveways, and yards is displayed in Figure 2. The roof area is represented by the indirectly connected impervious area, driveway by the directly connected impervious area, front yard by the connecting pervious area, and non-draining yards by the non-contributing areas. All remaining areas are represented by directly connected pervious areas using the KINEROS2 urban element. For GI practices, a retention basin can be represented by the retention basin area in KINEROS2 with an associated retention volume and hydraulic conductivity, a permeable driveway can be represented using the directly connected impervious flow area by specifying a hydraulic conductivity, and rainwater harvesting can be represented by specifying a cistern size to capture runoff from the indirectly connected impervious area. The user specified flow route in the AGWA Urban tool is converted to a KINEROS2 representation as shown in Figure 1. Although the flow route is drawn on the parcels, KINEROS2 assumes that each parcel contributes overland flow to the street half, and the subsequent runoff follows the specified flow route along the street.

**FIGURE 3 |:**
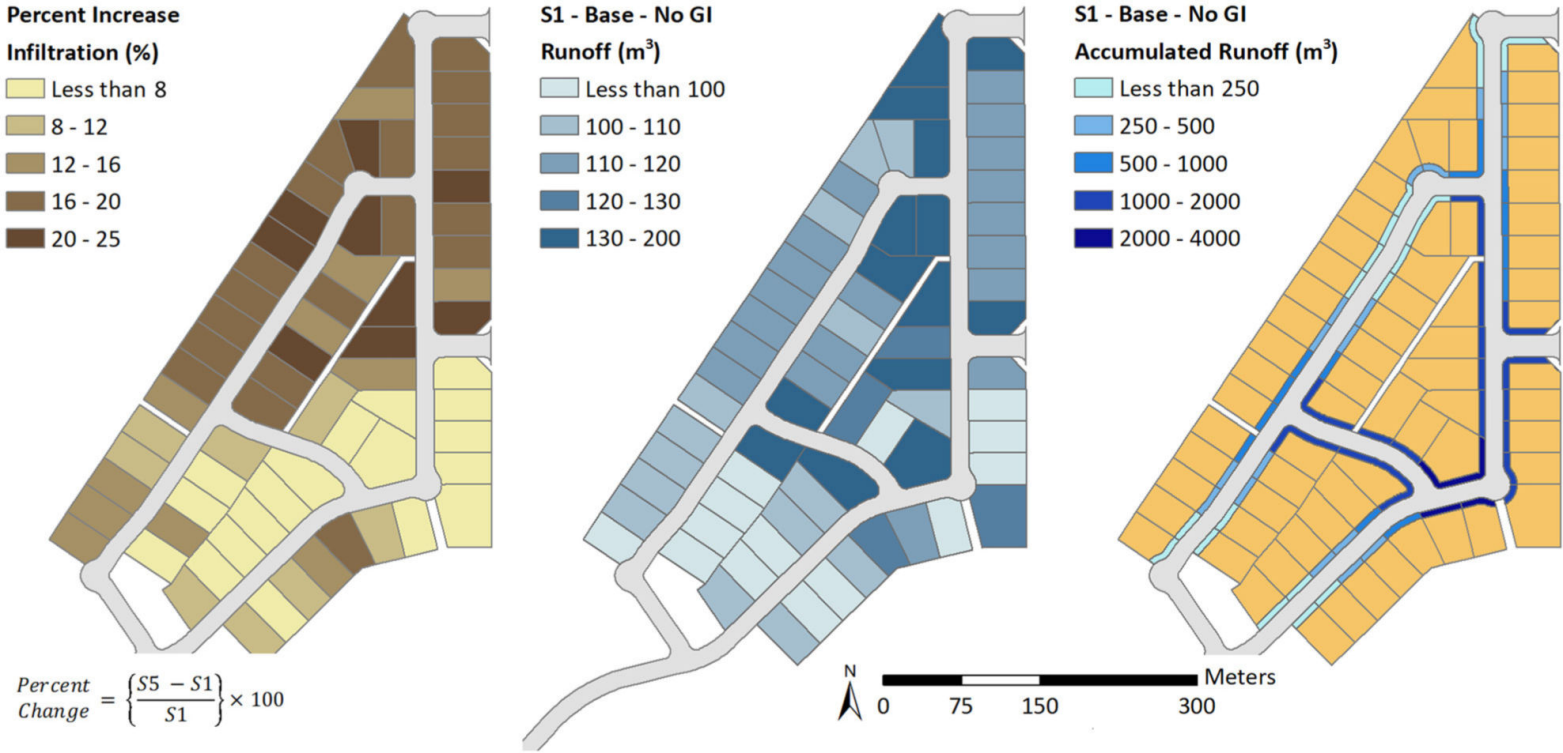
Visualization of results using the AGWA Urban tool with percent change in infiltration volumes between no GI practices in S1 and all GI practices in S5 **(Left)**, runoff volumes for S1 **(Center)**, and accumulated runoff from parcels and streets routed along the streets for S1 **(Right)**.

**FIGURE 4 |:**
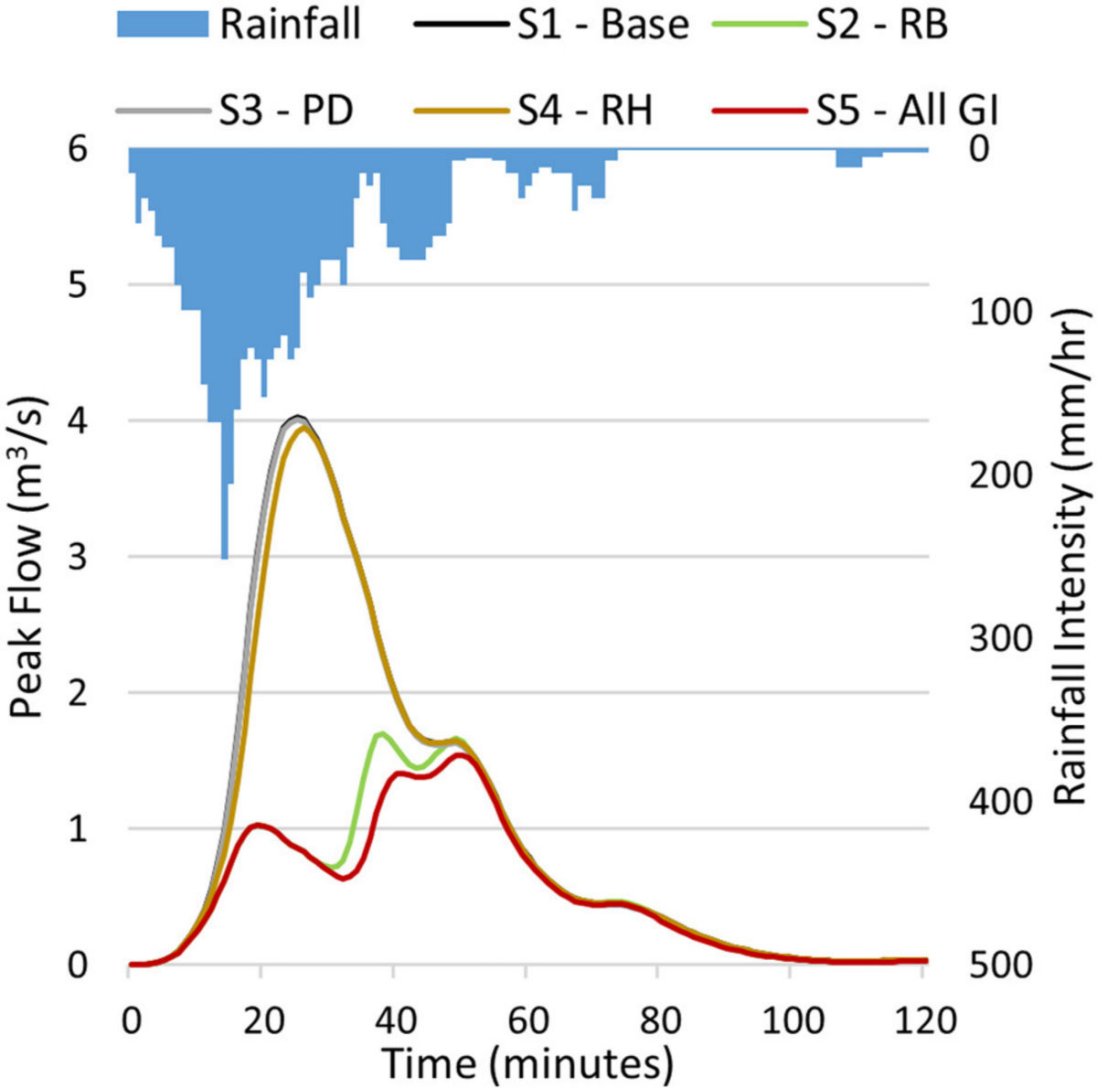
Hydrographs at the watershed outlet for the five scenarios for the July 11, 2014 event. S2-RB, with retention basin; S3-PD, with permeable driveways; S4-RH, with rainwater harvesting.

The AGWA Urban tool is capable of creating runoff, infiltration, and accumulated runoff maps (Figure 3). These maps can show absolute or percent difference in volumes between simulations for each individual parcel, demonstrated with percent increase in infiltration (Figure 3, left) from the S1 scenario (no GI) to the S5 scenario (all GI practices). Runoff volumes from a single scenario may also be displayed for each individual parcel, demonstrated with runoff from the S1 scenario (Figure 3, center). The accumulated runoff map (Figure 3, right) for scenario S1 (no GI) shows the cumulative runoff volume generated by the parcels and the streets combined as stormwater follows down the flow route toward the urban watershed outlet. Hydrographs can also be viewed, or exported from the AGWA Urban tool, for each parcel or at the urban watershed outlet (Figure 4).

### AGWA Urban Tool Validation

**FIGURE 5 |:**
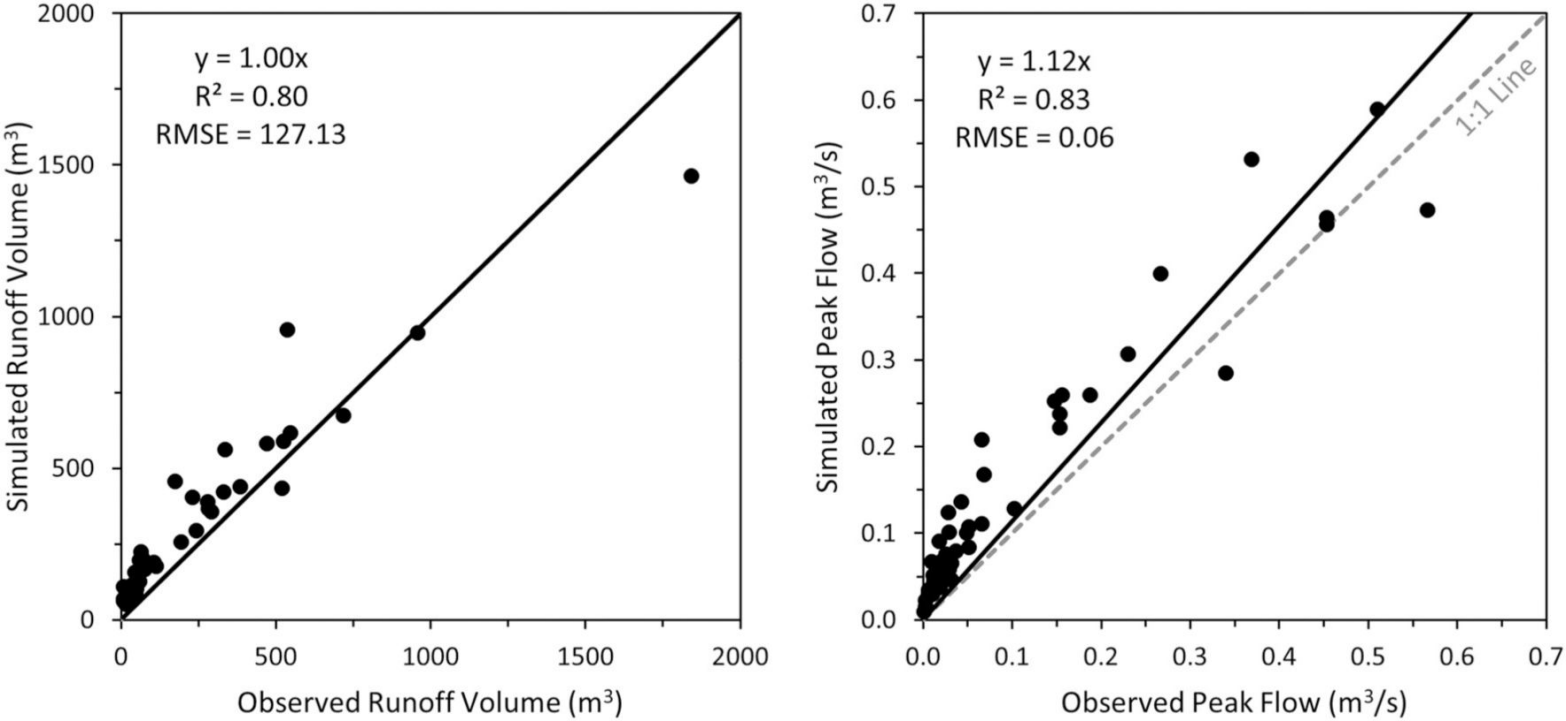
AGWA Urban tool validation results. Comparison of observed to simulated values for runoff volumes **(Left)** and peak flows **(Right)** at the watershed outlet.

Runoff volumes and peak flows simulated by the AGWA Urban tool and KINEROS2 model are compared with observed values in Figure 5. Regression equation slopes of 1.00 for runoff volumes and 1.12 for peak flows are indicative of good model performance. Root Mean Square Error (RMSE) was calculated for both runoff volumes and peak flows using Equation (1), where *n* is the number of events, and *S*_*i*_ and *O*_*i*_ represent the simulated and observed results for each event *i*.

(1) $$RMSE=\sqrt{\frac{\sum_{i=1}^{n}{\left(S_{i}-O_{i}\right)}^{2}}{n}}$$

Comparison of the runoff volumes results in an RMSE of 127.13 m^3^ (difference values ranging from −78.12 to 420.07), and peak flows results in an RMSE value of 0.06 m^3^/s (difference values ranging from −0.10 to 0.15) indicating a good fit of the model.

### Roof Runoff Analysis

Simulation results show an annual roof runoff capture of 7,078 m^3^ from all 66 parcels averaged over a period of 10 years. The average annual rainfall volume on all 66 parcels for the same period equals 47,280 m^3^. This means that the roofs are able to capture 15% of the total rainfall volume per year, for all the parcels combined, averaged over a 10-year period. The total roof area for all 66 parcels equals 25,085 m^2^, resulting in 28 cm of average annual depth of rainfall captured per unit area of the roofs for the La Terraza watershed.

Each house in the La Terraza urban watershed has the potential to capture an average of approximately 107 m^3^ of rainfall annually. Cochise County has a domestic per capita water usage of 0.41 m^3^/day that amounts to approximately 150 m^3^/year (Maupin et al., 2014). Thus, roof rainwater harvesting has a capacity to provide for almost 70% of the domestic annual per capita water use, assuming constant water use every month.

**FIGURE 6 |:**
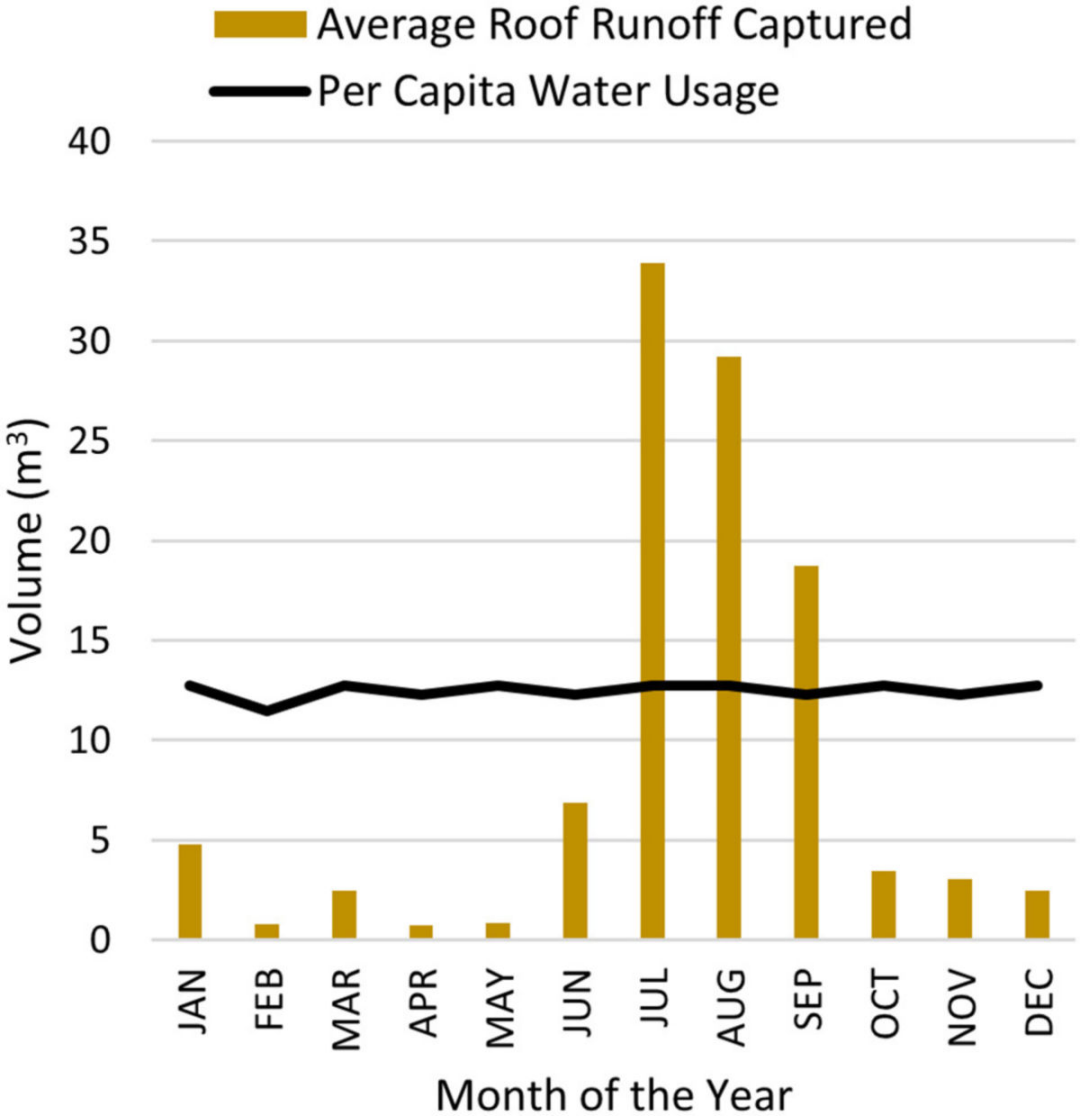
Average yearly roof runoff captured by a cistern and monthly per capita water usage for Cochise County estimated from a daily per capita usage rate of 0.41 m^3^/day.

Figure 6 shows monthly average volumes of roof runoff captured as compared to the monthly per capita water usage. Approximately 44 m^3^ of average roof runoff can be available as surplus water after household use in the monsoon months of June, July, August and September, every year.

### Case Study Scenarios

Table 2 summarize the average monthly runoff volumes at the watershed outlet for the five scenarios described in Table 1. Percent change in monthly runoff volumes were calculated using Equation (2), where S1 is the base scenario without GI practices, and *n =* 2, 3, 4, and 5 represent the four scenarios with GI practices, respectively.

(2) $$Percent\;Change\;=\left(\frac{Sn-S1}{S1}\right)\;\times \;100$$

For the simulated GI designs, retention basins in S2, and a combination of all GI practices in S5 have the highest reduction in average monthly runoff volumes at the watershed outlet, followed by permeable pavements in S3, and rainwater harvesting in S4, respectively. Scenarios S2 and S5 have identical results except for the month of July, when runoff volume for S5 is lower than S2. Percent reductions are greatest during the monsoon months as compared to the rest of the year for scenarios S2, S4, and S5. In contrast, S3 has lower percent reductions during the monsoon months. In summary, scenarios S2, S3, S4, and S5 show average volume reductions of 30, 14, 6, and 30%, respectively when compared to S1.

Further investigation of the near identical results of scenarios S2 and S5 revealed that the retention basins on each parcel had the capacity to retain and infiltrate all of the runoff generated from the respective parcel for most events. As a result, all runoff at the watershed outlet can be accounted for by rainfall excess generated by streets only.

One rainfall event on July 11, 2014, however, simulated lower runoff volumes at the watershed outlet for S5 as compared to S2. This rainfall event had a total depth of 83 mm over a 190-min duration amounting to a volume of 11,600 m^3^ over the entire watershed. The mean for all 787 events was 600 m^3^ (with a standard deviation of 1,006 m^3^) indicating that the July 11, 2014 event was indeed an outlier. This event compares to a 3-h, 200-year recurrence interval event from NOAA Atlas 14’s point precipitation frequency estimates for the Sierra Vista station. The July 11, 2014 event was the only event within the 10-year period capable of generating runoff from the parcels with the designed GI practices. The combination of retention basins, permeable driveways and the harvesting cistern in S5 was capable of capturing and reducing the parcel generated runoff, explaining the lower runoff volume for S5 as compared to S2 for the month of July. This event was a good indicator of the ability of GI to mitigate flood risks for higher recurrence interval events. Figure 4 shows a comparison of peak flow hydrographs for all five scenarios for this event at the watershed outlet. Scenario S5 has the highest peak attenuation followed by S2. The second peak in the hydrograph (around 37min into the event) for S2 and S5 is due to the retention basins exceeding their retention capacity and overflowing onto the streets. The smaller permeable driveway areas in S3 contribute very little to infiltration, and result in peak flows similar to the base scenario S1. Similarly, the 3.78m^3^ cistern on every parcel in S4, only manages to capture a small portion of the rainfall from the roofs of the houses.

**FIGURE 7 |:**
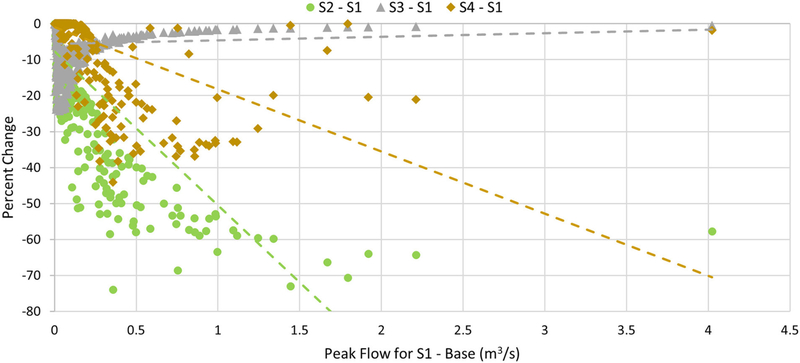
Trends in percent reduction of peak flows for each of the GI scenarios (S2, with retention basins; S3, with permeable driveways; and S4, with rainwater harvesting) as compared to S1 (without any GI practices) peak flows.

Percent change in peak flows at the watershed outlet for each of 787 rainfall events was calculated using Equation (2). Figure 7 plots the percent reduction against peak flows for S1 to identify trends in the ability of GI practices to affect peak flows at the watershed outlet. S2 has overall higher percent reductions in peak flows as compared to S3 and S4 due to amount of retention volume available, especially for larger S1 peak flows. S3 shows better peak flow reduction as compared to S4 for S1 peak flows lower than 0.2 m^3^/s. However, for higher S1 peak flows, peak flow reduction for S3 decreases. It is also important to note that majority of the storms generate peak flows less than 1.0m^3^/s, and peak flows do not exceed 2.25 m^3^/s, except for the July 11, 2014 event.

## DISCUSSION

### The AGWA Urban Tool

The AGWA Urban tool provides a user-friendly method to setup and execute the KINEROS2 model. Parcel data is commonly available from the local city or county office. With the availability of high-resolution imagery, each parcel can be digitized to extract roof, driveways and yard areas. Stormwater flow routes for an urban watershed can be digitized based on slope calculated from terrestrial airborne surveys, using Light Detection and Ranging (LiDAR), Structure from Motion (SfM), or another alternative. For the purpose of this study, the SSURGO dataset was utilized to extract soil parameters. However, in order to consider changes in soil properties due to compaction and landscape modifications, field measurements can be converted into spatial data for use in the GIS. Precipitation data is usually measured using a number of rain gauges depending on the size of the watershed. Additionally, estimated ground accumulated rainfall is available through precipitation products derived from Next Generation Weather Radar (NEXRAD) system from the NOAA’s National Centers for Environmental Information (NCEI) for a number of locations in the USA. The availability of these spatial datasets and the ability to import them into the GIS, helps simplify and speed up the process of setting up the KINEROS2 model using the AGWA Urban tool.

The use of individual parcels to define the smallest modeling urban element in KINEROS2 enables a fine-scale simulation of urban hydrology. The nine urban overland areas can simulate a number of combinations of flow-off and flow-on areas at a detailed scale. It is important to note that the KINEROS2 representation of a parcel is always assumed rectangular. As a result, for non-rectangular shaped parcels, roofs, driveways, and yards, a distortion of physical reality must be expected in the representation.

A combination of the detailed parcel representation and the ability to simulate sub-hour time steps for rainfall events enables the KINEROS2 model to represent the physical processes of infiltration and runoff for each parcel, and their responses to rainfall variability within an event. This gives a better understanding of the role of each individual parcel and its GI practices, as well as its contribution to the overall hydrology of the urban watershed.

The infiltration and runoff maps from Figure 3 can help identify parcels with lower infiltration, and that generate higher runoff. These parcels can then be potential targets for installation of GI practices to capture rainwater using cisterns, or encourage infiltration by installing retention basins or permeable driveways. Potential impacts of GI applications can be analyzed for before-after scenarios using the differencing capability available in the AGWA Urban tool. Potential flood-prone streets and intersections can be identified using the accumulated runoff map (Figure 3, right). Street GI practices, such as curbside retention basins, swales, or traffic circles can then be designed to mitigate flood at these locations.

### AGWA Urban Tool Validation

Validation results indicate that runoff volumes, on average, are predicted well by KINEROS2, while peak flows are generally over-predicted for the validation configuration. Over-prediction of volumes and peak flows may be due to a lower time of concentration on the parcels due to misrepresentation of physical reality. This misrepresentation may be a result of the assumption that all front yards (CP) and non-contributing (NC) areas on each parcel are equal to 10% of the lot area. For example, a parcel may have a smaller simulated pervious area (CP) that captures and retains roof runoff (ICI), as compared to the physical reality. As a result, the simulated volume of runoff reaching the streets is higher, resulting in higher peak flows for that particular event. Another reason could be that some of the parcels may have a larger non-contributing area, such as a backyard that stores and infiltrates most of the runoff, instead of contributing as overland flow to the street. In the La Terraza urban watershed, the yard area can be up to 4.5 times the footprint of the living area. This may result in lower observed runoff volumes and peak flows at the outlet, when compared to the simulated results.

### Roof Runoff Analysis

Roof runoff analysis estimated that on an average, 70% of the roof runoff captured every year was available for household use. Monthly rainfall volumes, averaged over a period of 10 years show seasonal trends with more rainfall occurring in the months of June, July, August, and September (Table 2). These months represent the monsoon season with short duration, high intensity thunderstorms caused due to convection. Rainfall volumes are lower in the winter months and are characterized by long duration, low intensity storms due to frontal lifting (Sheppard et al., 2002; Brooks et al., 2003). The roof runoff capture potential is high for the monsoon season, when more water is available via rainfall (Figure 6). This harvested rainwater can be stored and utilized during the drier months of the year. In a semi-arid climate, this harvested rainwater can augment scarce water supply sources in order to meet daily household water needs.

**FIGURE 8 |:**
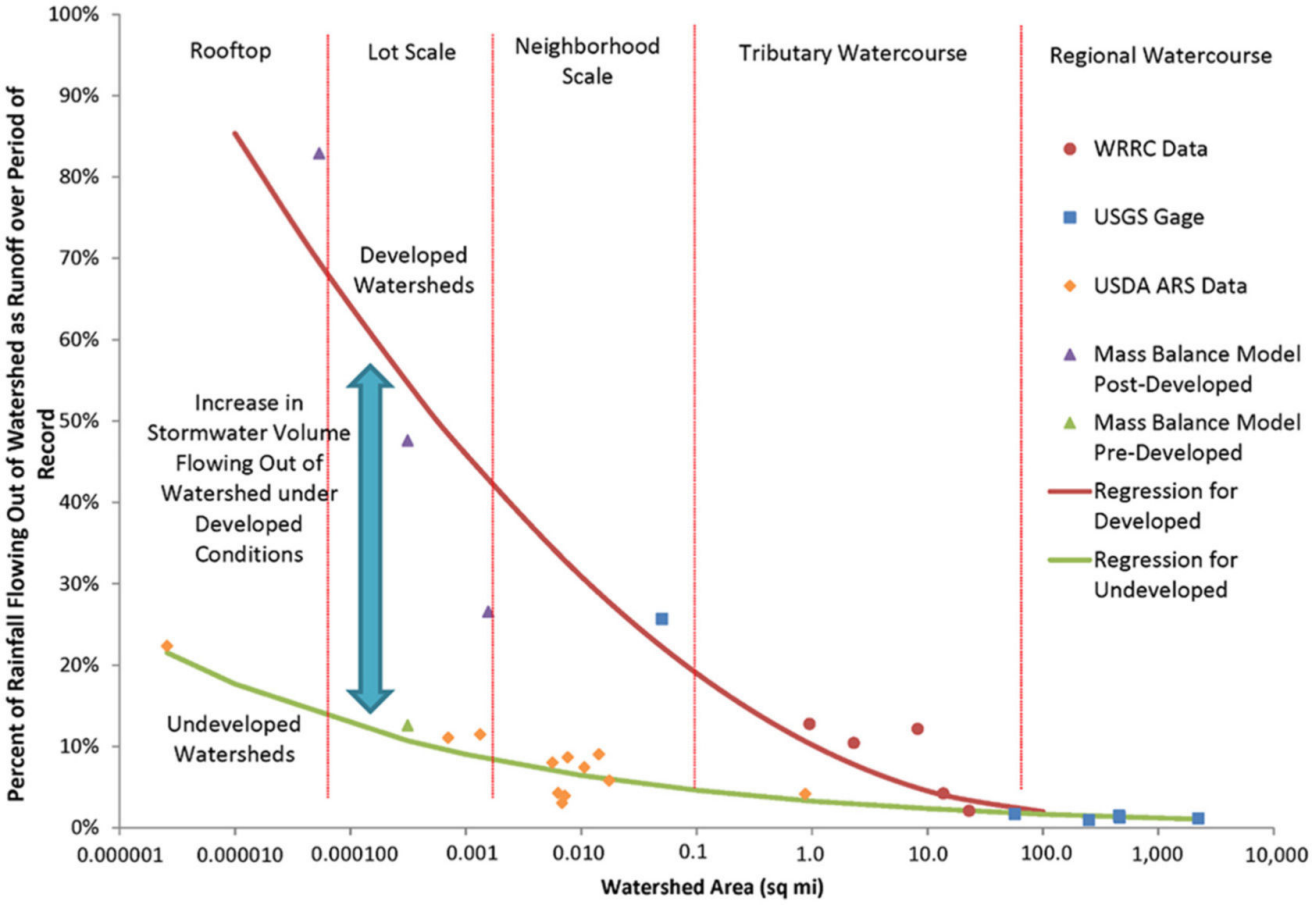
Harvestable rainwater at different watershed scales (City of Tucson Pima Country, 2009). Figure created by Dr. Evan Canfield (used with permission).

City of Tucson Pima Country (2009) conducted an evaluation of the use of stormwater and rainwater as a supplemental water source. This study looked at potential harvestable rainwater at different scales ranging from a parcel to a tributary watercourse in the semi-arid climate of Southern Arizona. Figure 8 reveals that the greatest opportunity to harvest rainwater is at the parcel scale, with roofs being the primary contributor to rainfall excess, especially in the semiarid region of Southwestern USA. This supports that rainwater harvesting is a good alternate source of water for irrigation, toilet flushing, and consumption (Thomas et al., 2014; Silva et al., 2015; Campisano et al., 2017). However, roof runoff could be a source of chemical or microbial pollutants and could have health risks from its use as drinking water (Lye, 2009; Gwenzi et al., 2015). Hence, treatment of this water is essential before potable household use.

### Case Study Scenarios

Case study scenarios indicated that retention basins were effective at volume and peak flow reduction as compared to permeable driveways and rainwater harvesting, particularly during the monsoon months. The monsoon months result in larger rainfall volumes, thereby causing larger rainfall excess available for capture. Permeable driveways showed better volume and peak flow reductions for smaller events during the non-monsoon months of the year. During these months, rainfall intensity is generally low and could possibly be closer to the infiltration capacity of the permeable driveway. The capability of rainwater harvesting cisterns to reduce runoff volumes and peak flows is largely limited by the size of the cistern, and by the proportion of roof area available vs. the total parcel area. With larger cisterns, and roof areas, higher runoff volumes and peak reduction should be expected.

GI design can play an important role in determining effectiveness in runoff and peak flow reduction (Liu et al., 2015). It is important to note that the GI designs used in this study, were “back-of-the-envelope” values in order to represent possible GI installation due to lack of physical implementations. For example, for an average parcel size of 1,780 m^2^, a retention basin would have a surface area of 178m^2^ and depth of 0.3m, with a retention volume of 53.4 m^3^. This retention volume may be considered on the higher side, possibly representing a best-case scenario, which may or may not be a practical implementation. However, a 3.785 m^3^ cistern is a reasonable assumption for implementation at a housing parcel level. Additionally, the case study assumed that basins and cisterns were empty at the start of each event, which may not be the case in real-world scenarios. If a cistern is not emptied between events, it may result in higher roof generated runoff. As a result, this assumption can lead to an under-estimation of overall runoff exiting from a parcel.

Initial soil saturation in between rainfall events can affect predicted runoff in the KINEROS2 model (Yatheendradas et al., 2008). Therefore, these case study results are limited by the assumption of a 0.2 initial soil saturation. This value represents the soil saturation at permanent wilting point for sandy loam soils commonly found in this region (Woolhiser et al., 1990), simulating a best-case infiltration scenario. With variable initial soil saturation in between events, uncertainty in simulated runoff can be expected. In general, higher initial soil saturation will result in a decrease in the infiltration capacity of the soil, thereby resulting in larger simulated runoff and peak flows. In the KINEROS2 urban element, initial soil saturation only affects areas modeled as directly connected pervious (DCP) and connecting pervious (CP). KINEROS2 assumes a constant seepage rate for retention basins and permeable driveways. Thus, initial soil saturation does not influence the amount of rainwater harvested off the roofs (scenario S4), nor the amount of rainfall directly infiltrated in retention basins (S2) or permeable driveways (S3). However, DCP and CP contribute runoff to the retention basins as shown in Figure 2. As a result, initial soil saturation in these pervious areas will affect the overall runoff and peak flows for scenario S3 and S5, simulating the retention basins.

Other studies show similar results in volume and peak flow reductions using different combinations of GI practices (Lloyd et al., 2002; Ahiablame et al., 2013; Palla and Gnecco, 2015; Ahiablame and Shakya, 2016; Eckart et al., 2017; Fry and Maxwell, 2017; Hu et al., 2017). These studies conclude that there is scope for volume and peak flow reduction at various watershed scales using GI practices. The design criteria of the GI practices largely determines their effectiveness. Spatial variability in GI implementation could also influence level of effectiveness. These studies also provide an insight in the potential designs and locations of GI practices for their respective watersheds. Future research should be guided toward spatial variability of GI implementation, to understand its effects on volume and peak flow reduction.

## CONCLUSIONS

The AGWA Urban tool provides an easy-to-use GIS framework to prepare and execute the KINEROS2 model in order to simulate urban hydrology and green infrastructure. The KINEROS2 model is able to model the built environment with the help of the urban element that utilizes nine overland flow areas to simulate flow-on and flow-off processes for different areas. For a typical urban parcel, the roof, driveway, yards, and swimming pools, can be represented by indirectly connected impervious (ICI), directly connected impervious (DCI), directly connected pervious (DCP) and connecting pervious (CP), and non-contributing (NC) flow areas, respectively. GI practices such as retention basins, permeable driveways, and rainwater harvesting cisterns can be represented using the RB, DCI, and ICI urban element components respectively, by providing appropriate parameters. A combination of multiple urban elements can define an urban watershed.

The AGWA urban tool utilizes spatial data, such as parcels, streets, land cover, precipitation, and soils, to extract input parameters required by the KINEROS2 model. Each individual parcel can be represented using the KINEROS2 urban element (Figure 2), and can be associated with a unique set of parameters. The AGWA Urban tool is able to extract these parameters from input spatial datasets to prepare and execute the KINEROS2 model. The tool also compiles and synthesizes the simulation results for visualization in the form of runoff and infiltration volume maps (Figure 3), as well as peak flow hydrographs (Figure 4). Infiltration and runoff volume maps can help identify parcels with lower infiltration and higher runoff volumes as potential GI implementation sites. Percent change analyses can be conducted to understand the impacts of GI implementation on a parcel-by-parcel basis.

The AGWA Urban tool was validated using 47 rainfall events on the La Terraza subdivision in Sierra Vista, Arizona. Sixty-six parcels were identified and modeled as an urban watershed. Simulated runoff volumes and peak flows were compared with observed values at the outlet of the watershed (Figure 5). The regression equation for the runoff volumes comparison yielded a slope of 1.00 with an *R*^2^ value of 0.80, and yielded a slope of 1.12 with an *R*^2^ value of 0.83 for the peak flow comparison. In general, runoff volumes were well predicted, but peak flows were over-predicted by the model.

Seven-hundred and eighty-seven rainfall events were simulated on the same urban watershed over a period of 10 years, from January 2006 to December 2015 to analyze the potential to capture roof runoff via harvesting cisterns. Simulation results indicated a 15% capture of the average monthly rainfall volume with a volume capture rate of 0.28 m^3^/m^2^. Additionally, roof rainwater harvesting has the potential to provide up to 70% of the domestic annual per capita water use in Cochise County, Arizona.

Five scenarios (Table 1) were simulated to analyze the impact and effectiveness of retention basins (S2, 53.31 m^3^ average volume), permeable driveways (S3), and rainwater harvesting (S4, 3.78 m^3^ capacity) on runoff volumes and peak flows at the watershed outlet. Retention basins in S2 reduced runoff volumes by almost 30%, permeable driveways in S3 reduced runoff volumes by 14%, and rainwater harvesting in S4 was successful in capturing around 6% of the runoff volume. A combination of all three GI practices in S5 resulted in identical volume and peak flow reduction as S2. Seasonal trends were also observed for all scenarios, with an increase in effectiveness in runoff reduction for S2, S4, and S5 for the monsoon months of June, July, August, and September, when high-intensity rainfall events are observed. However, permeable driveways in S3 showed lower runoff volume reduction for larger events during the monsoon season.

For 786 rainfall events, the street network was the sole contributor to runoff and peak flow at the watershed outlet for S2 and S5 simulations. This was because the retention basin was capable of retaining all of the overland runoff originating on the parcel, representing a best-case scenario. A rainfall event for July 11, 2014 was the only event for which the retention basins were overwhelmed, and the parcels contributed overland runoff to the street network. The magnitude of this rainfall event compares to a 3-h 200-year recurrence interval event determined from NOAA Atlas 14’s point precipitation estimates. For this event, scenarios S2 and S5 resulted in higher peak flow reduction at the watershed outlet as compared to S3 and S4 (Figure 4).

Analysis of all peak flows at the watershed outlet for the 787 events revealed that S5 had the highest overall peak flow reduction followed by S2, S3, and S4, when compared to the base scenario S1 without any GI practices (Figure 7). Interestingly, permeable driveways in S3 were more effective than rainwater harvesting in S4 for lowering peak flows compared to no GI practices in S1, and percent reduction decreased as the magnitude of S1 peak flows increased. Limited driveway sizes and the divergence between rainfall intensity and infiltration capacity at larger events restrict the infiltration potential, which explains this trend.

This study primarily focused on the potential of lot-level GI practices to capture rainfall excess in order to augment water supply in semi-arid regions. Roofs can have the highest potential to capture rainwater for domestic use. Rainwater captured during the larger monsoon events can be stored for use during the drier months. We can conclude from our analysis, that appropriate GI designs can be extremely effective at capturing rainfall excess on site for smaller events. It is important to note that these conclusions are based on specific GI designs, and will require future research to compare the impact of different GI designs for each of the practices, for a variety of return period events. However, we also advocate the use of the AGWA Urban tool for exploring GI construction options in arid and semi-arid environments, for the purpose of water conservation and reduced catastrophic surface flow during storm events. With the success of the various modeling scenarios presented in this study, we recommend the use of the AGWA Urban tool for modeling hydrology and GI practices in the built environment.

## Figures and Tables

**TABLE 1 |:** Description of the five case study scenarios.

Scenario	GI practice	Description
S1	Base - No GI practices	Validated model without any GI practices
S2	Retention Basin (RB)	S1 with retention basin on all 66 parcels. Retention basin area equals 10% of the parcel area, depth equals 0.3 m, and hydraulic conductivity of 8.3 mm/h. Average retention basin capacity equals 53.41 m^3^.
S3	Permeable Driveways (PD)	S1 with all 66 driveways considered permeable with a hydraulic conductivity of 8.3 mm/h
S4	Rainwater Harvesting (RH)	S1 with a 3.78 m^3^ cistern on each of the 66 parcels capturing roof runoff
S5	All GI practices	S1 with GI designs from S2, S3, and S4 in combination on each of the 66 parcels

**TABLE 2 |:** Monthly runoff volumes at the watershed outlet for the five case study scenarios averaged over a period of 10 years (January 2006 to December 2015) and percent reduction in monthly runoff volumes for the scenarios as compared to the S1 base scenario (no GI) for the same period.

Month	Rainfall (m^3^)	Runoff volumes at watershed outlet (m^3^) and volume reduction compared to S1 (%)								
		S1–Base No GI	S2–RB		S3–PD		S4–RH		S5–All GI	
		m^3^	m^3^	%	m^3^	%	m^3^	%	m^3^	%
January	2,245	479	363	24	390	19	470	2	363	24
February	571	106	92	13	93	13	106	<1	92	13
March	1,349	271	218	20	229	16	264	3	218	20
April	392	77	63	18	64	17	77	1	63	18
May	383	79	62	21	63	20	78	1	62	21
June	2,944	1,057	476	55	993	6	953	10	476	55
July	14,274	4,706	2552	46	4,368	7	4,013	15	2,519	46
August	12,448	3,958	2013	49	3,669	7	3,344	16	2,013	49
September	8,064	2,287	1304	43	2,058	10	1,968	14	1,304	43
October	1,659	361	268	26	307	15	333	8	268	26
November	1,552	325	251	23	269	17	318	2	251	23
December	1,399	277	226	18	231	16	273	1	226	18
Average	3,940	1,165	658	30	1,061	14	1,016	6	655	30
